# Metastatic adenoid cystic carcinoma of the lacrimal gland with parotid and pulmonary involvement: a rare case report

**DOI:** 10.1093/jscr/rjaf216

**Published:** 2025-04-15

**Authors:** Elias Edward Lahham, Dana Abu Naji, Sally Diab, Harun Awadallah, Shorok Mahajna, Adam Dacca, Mohand Abulihya, Jalal Qawasmeh

**Affiliations:** Department of Radiation Oncology, Augusta Victoria Hospital, Martin Buber Jerusalem 9119101, East Jerusalem, Palestinian Authority, Palestine; Faculty of Medicine, Al-Quds University, Main Campus, Abu Dis, PO Box 89, Palestine; Faculty of Medicine, Al-Quds University, Main Campus, Abu Dis, PO Box 89, Palestine; Faculty of Medicine, Al-Quds University, Main Campus, Abu Dis, PO Box 89, Palestine; Faculty of Medicine, Al-Quds University, Main Campus, Abu Dis, PO Box 89, Palestine; Faculty of Medicine, Al-Quds University, Main Campus, Abu Dis, PO Box 89, Palestine; Department of Pathology, Augusta Victoria Hospital, Martin Buber Jerusalem 9119101, East Jerusalem, Palestinian Authority, Palestine; Department of Radiation Oncology, Augusta Victoria Hospital, Martin Buber Jerusalem 9119101, East Jerusalem, Palestinian Authority, Palestine

**Keywords:** adenoid cystic carcinoma (ACC), lacrimal gland, parotid gland, metastasis

## Abstract

Adenoid cystic carcinoma (ACC) is an uncommon tumor that arises mainly in the head and neck area. It represents <1% of all head and neck malignancies and about 4–10% of all salivary gland tumors. This study presents a 27-year-old patient who was found to have ACC of the lacrimal gland and was treated with surgical resection and adjuvant radiotherapy, came back with metastatic disease to the parotid gland and the lungs. This review explores the clinical, radiographic, and pathological characteristics of these uncommon malignant tumors, along with available treatment approaches.

## Introduction

Adenoid cystic carcinoma (ACC) is an aggressive epithelial tumor commonly seen in the esophagus, bronchial glands, the major salivary glands, and the skin. ACC is the lacrimal gland's most frequently occurring malignant histological type, comprising 25% to 40% of all epithelial tumors in this region. [1], it has a high rate of recurrence and metastasis despite aggressive treatment. The tumor is known for its tendency to recur, with a review of 29 ACC case reports revealing that 54% of cases developed distant metastases over an average follow-up of three years. [2] Metastasis from lacrimal gland adenoid cystic carcinoma often affects the lungs, kidneys, brain, and liver. Parotid gland metastasis is rare, with limited data available. Our case focuses on diagnosis and treatment, highlighting challenges in optimal management. Conservative surgery and external beam radiation are commonly recommended for primary tumors, but parotid metastasis treatment remains challenging.

## Case presentation

A 27-year-old female patient with no significant medical history presented with a painless lump over the lateral canthus of her left eye. Over several months, the swelling enlarged, causing intermittent, localized pain without numbness, facial palsy, or visual changes. Examination revealed a well-oriented patient with normal cranial nerves and a non-tender left lateral eye swelling. CT scan showed a 3 × 3 × 2.7 cm soft tissue mass with destruction of the lateral orbital wall, while the optic nerve and neck lymph nodes were normal. Biopsy confirmed adenoid cystic carcinoma (ACC).

**Figure 1:**
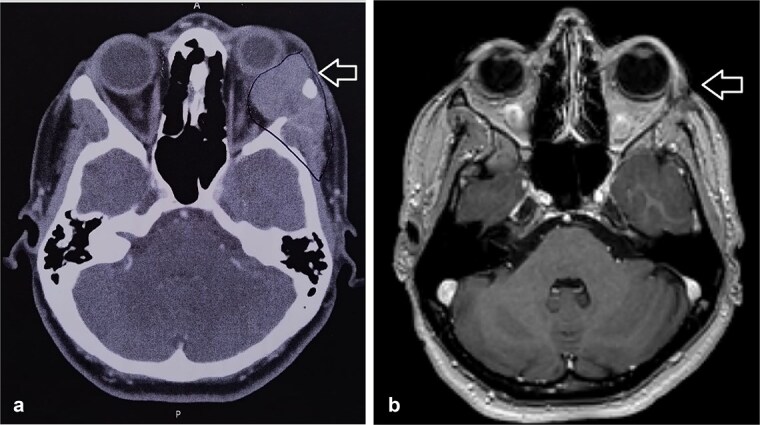
(a) CT scan showed left orbital mass with both solid enhancing and cystic components. The epicenter of the mass appears to be in the left lacrimal gland. (b) MRI postoperative that showed complete tumor excision.

The patient underwent left orbital transcanthal craniotomy and orbitotomy with resection of the left retro-bulbar tumor. Left neck dissection was conducted with excision of lymph nodes for staging. Assessment of the margin was not performed due to the fragmented nature of the bone, so the patient underwent adjuvant radiotherapy (Fig. 1).

**Figure 2:**
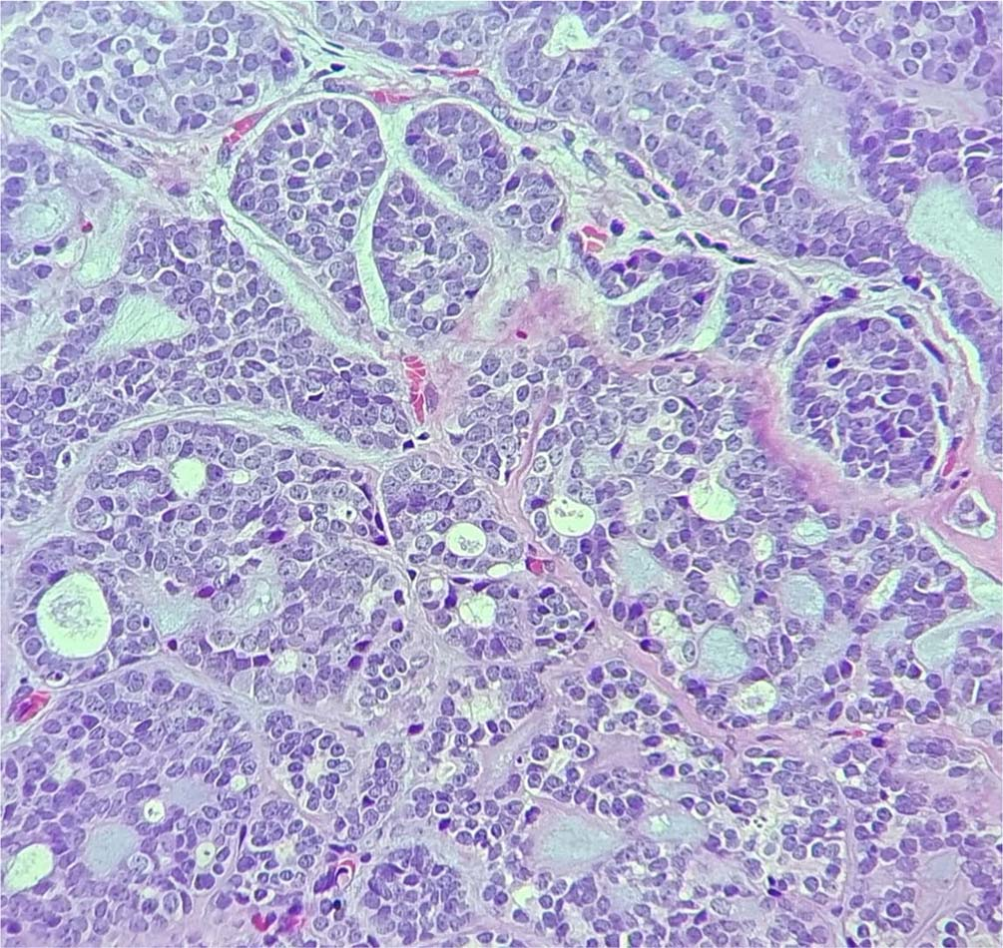
The tumor is composed of dual cell populations of basaloid epithelial cells and myoepithelial cells, arranged in characteristic cribriform and tubular growth patterns.

Histopathologic examination showed ACC displaying multiple cribriform structures composed of epithelial and basal/myoepithelial cells (Fig. 2). Molecular profiling indicated negative estrogen receptor, progesterone receptor, HER2, and PDL-1.

**Figure 3:**
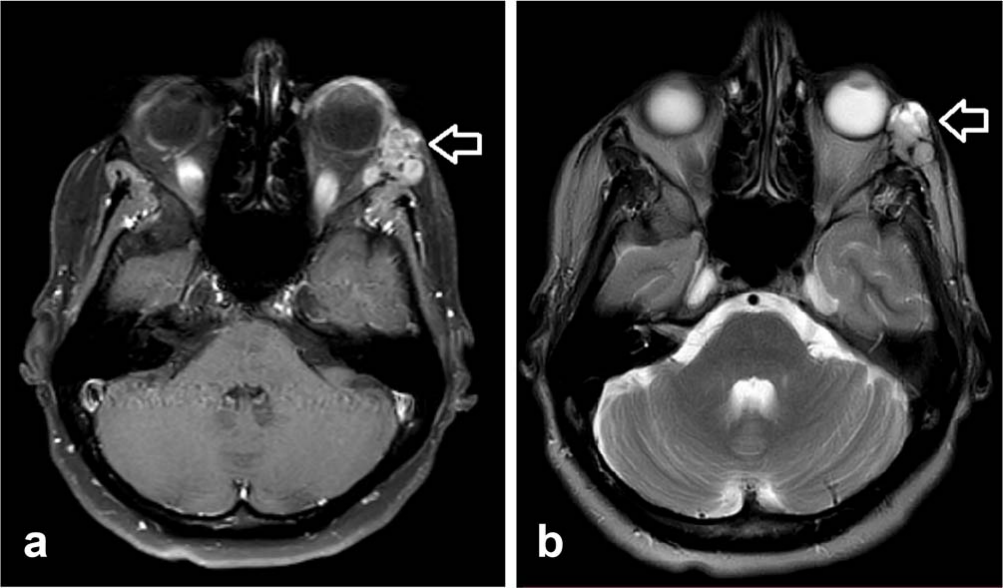
Orbit MRI showed lobulated well-defined lesion, hyperintense to intermediate on T2W (a), hypointense on T1W (b) structures with cystic changes measuring 2 × 1.5 cm and 2 × 1.2 cm. After IV contrast administration they showed heterogeneous moderate enhancement.

She was kept on follow-up for four years with clinical exams and MR imaging until she felt a palpable mass at the left lower dental root with local disease recurrence on orbit MRI (Fig. 3). An excisional biopsy from the submandibular mass revealed ACC, indicating metastatic disease. She was consequently given five cycles of paclitaxel and carboplatin chemotherapy.

**Figure 4:**
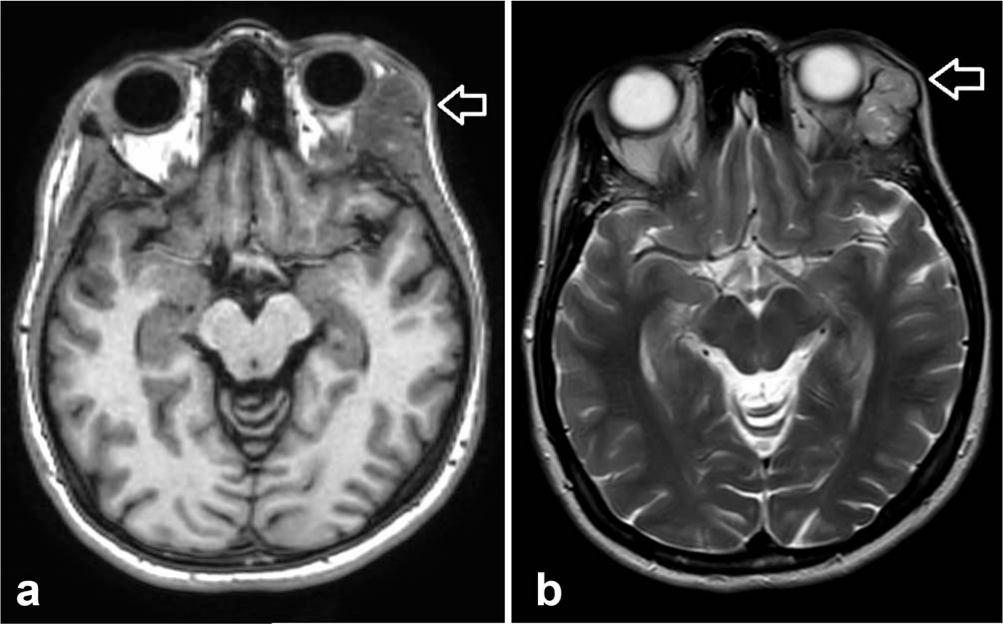
Orbit MRI showed a lobulated, well-defined lesion, hyperintense to intermediate on T2W (b) and hypointense on T1W (a) structures with cystic changes, measuring 2.3 × 1.9 cm and 2.6 × 1.6 cm, compared with the previous study, the lesions have increased in size.

**Figure 5:**
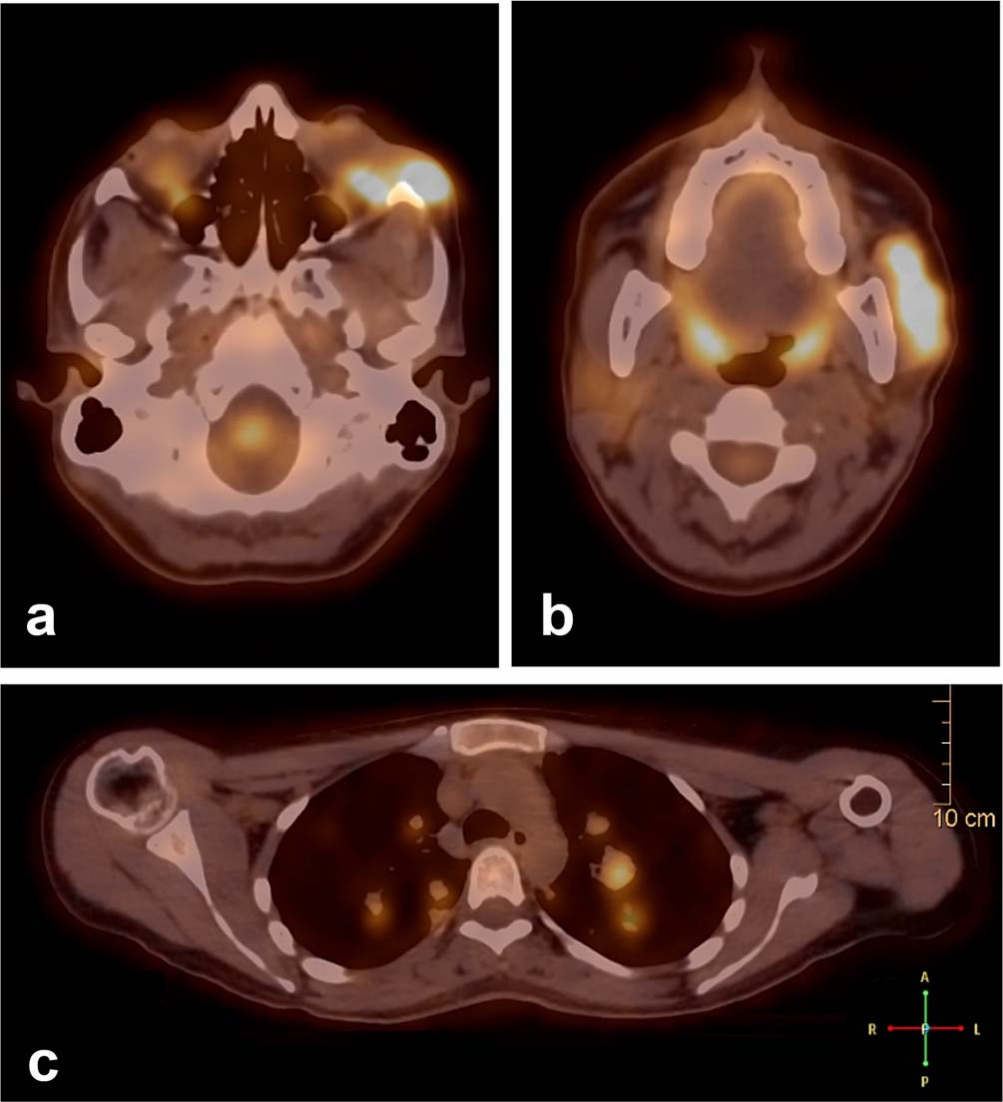
A PET scan revealed hypermetabolic malignant irregular soft tissue thickening within the lateral periorbital side of the left eye (a), indicating a recurrence of ACC of the left eye, and a new hypermetabolic irregular soft tissue mass in the left parotid gland (b). Numerous bilateral scattered pulmonary nodules were noted (c).

During follow-up, the patient complained of swelling at the left angle of the mandible and left lateral canthus. Orbit MRI showed local disease progression (Fig. 4). Ultrasonography of the left parotid region showed an irregular hypoechoic lesion in the left parotid gland. A PET scan revealed hypermetabolic malignant irregular soft tissue thickening within the lateral periorbital area of the left eye, consistent with a recurrence disease. Additionally, there is a new hypermetabolic irregular soft tissue mass in the left parotid gland with numerous bilateral scattered pulmonary nodules (Fig. 5). A biopsy from the left parotid gland confirmed the diagnosis of ACC, aligning with the primary tumor. The patient was diagnosed with lacrimal gland ACC with metastasis to the left parotid gland and the lungs. The Head and Neck multidisciplinary team proceeded with systemic chemotherapy using cisplatin and Navelbine. The patient started chemotherapy with good tolerance and clinical response and is now on regular follow-up with the oncology clinic.

## Discussion

ACC primarily originates from the epithelial cells of the lacrimal gland and exhibits histopathological similarities to ACC found in salivary glands. The key histological patterns—cribriform, tubular, and solid—provide diagnostic specificity, with the cribriform pattern being the most prevalent and linked to a more favorable prognosis compared to the solid subtype [3]. ACC of the lacrimal gland presents with proptosis, pain, and diplopia, showcasing aggressive behavior and parotid metastasis via lymphatic or hematogenous routes.

ACC is characterized by local recurrence and distant metastases, predominantly to the lungs. Ipsilateral parotid gland metastasis is rare because it is not a primary drainage or neural pathway for lacrimal gland tumors. There are different potential pathways of spread:

1. Lymphatic spread: The lacrimal gland drains to the preauricular and parotid lymph nodes, and ACC is known for early lymphatic infiltration, which might have established the pathway.
2. Perineural invasion: It is a hallmark of ACC. The tumor might have spread along the cranial nerves, particularly branches of the trigeminal nerve. This pathway could have facilitated direct migration.
3. Contiguous spread: Large tumors may extend through the lateral orbital wall to invade the parotid gland directly.
4. Hematogenous spread: It is more common for distant metastases like the lungs and the liver, the vascular connection between the orbit and the parotid could occasionally permit this route.

MRI is the preferred modality to detect bony invasion and perineural spread. ACC usually appears as T1 isointense and T2 hyperintense with enhancement . Our patient's orbit MRI showed a lobulated, well-defined lesion, hyperintense to intermediate on T2W and hypointense on T1W structures with cystic changes.

ACC treatment requires a multi-modality strategy because of its high recurrence rate nowadays, the most acceptable approach is orbital excision with radiation therapy and chemotherapy [4]. Orbital exenteration remains the cornerstone for achieving local disease control, particularly in cases with significant local invasion [5]. Selecting the optimal surgical strategy for these lesions is one of the most crucial management decisions, as it can significantly impact the patient's mortality and morbidity. A key factor influencing both progression-free survival and overall survival after surgery is the presence of residual tumor burden. Therefore, great care should be taken to reduce gross residual disease [6]. Given the low incidence of lymph node metastases (4% to 9%), it may not be required to dissect lymph nodes. [5, 6].

Worldwide, the extent of surgical resection (orbital exenteration versus eye-sparing surgery) remains a debated issue. Orbital exenteration has been the most performed surgery for lacrimal gland ACC, based on the belief that it enhances survival outcomes [7]. However, according to the research, orbital exenteration increases the likelihood of distant relapse and cancer-related death [7], highlighting the disease's locally invasive and metastatic characteristics.

Postoperative radiation therapy following surgical treatment is widely used as the standard treatment of advanced ACC especially if adequate surgical margins cannot be achieved, if there is bony structure invasion, or if there is intracranial invasion [8]. With its ability to deliver targeted doses, proton beam therapy has shown promise in improving local control while minimizing damage to adjacent tissues. One study found that less than 25% of patients who received postoperative radiotherapy, developed tumor recurrence [9]. Our patient had a recurrence of the disease later on, and metastasis to the ipsilateral parotid gland even though she underwent radiotherapy, which emphasizes the high recurrence rate of the disease and its ability to metastasize to different and uncommon sites.

This case can help clinicians recognize atypical metastatic patterns and tailor multimodal treatment approaches, and it highlights the need for comprehensive imaging to detect uncommon metastatic sites.

## Conclusion

ACC is a rare, aggressive epithelial tumor with a high recurrence and metastasis rate. This case emphasizes ACC's potential to metastasize to uncommon sites, such as the parotid gland. Early detection, multidisciplinary treatment, and comprehensive imaging are vital for managing atypical metastatic patterns and improving patient outcomes.

## Data Availability

The data used to support the findings of this study are included within the article.
