# Correction to: Metastatic adenoid cystic carcinoma of the lacrimal gland with parotid and pulmonary involvement: a rare case report

**DOI:** 10.1093/jscr/rjag362

**Published:** 2026-04-27

This is a correction to: Elias Edward Lahham, Dana Abu Naji, Sally Diab, Harun Awadallah, Shorok Mahajna, Adam Dacca, Mohand Abulihya, Jalal Qawasmeh, Metastatic adenoid cystic carcinoma of the lacrimal gland with parotid and pulmonary involvement: a rare case report, *Journal of Surgical Case Reports*, Volume 2025, Issue 4, April 2025, rjaf216, https://doi.org/10.1093/jscr/rjaf216

The second co-author's name has been corrected to read: “Dana Abu Naji”.

